# An exploration of new methods for metabolic syndrome examination by infrared thermography and knowledge mining

**DOI:** 10.1038/s41598-022-10422-6

**Published:** 2022-04-16

**Authors:** Bao-Hong Mi, Wen-Zheng Zhang, Yong-Hua Xiao, Wen-Xue Hong, Jia-Lin Song, Jian-Feng Tu, Bi-Yao Jiang, Chen Ye, Guang-Xia Shi

**Affiliations:** 1 School of Acupuncture-Moxibustion and Tuina, Beijing University of Chinese Medicine, Beijing, 100029 China; 2 Dongzhimen Hospital, Beijing University of Chinese Medicine, Beijing, 100010 China; 3 School of Electrical Engineering, Yanshan University, Qinhuangdao, 066004 Hebei China; 4 International Acupuncture Innovation Institute, Beijing University of Chinese Medicine, Beijing, 100029 China

**Keywords:** Metabolic syndrome, Mathematics and computing, Imaging and sensing, Infrared spectroscopy

## Abstract

Metabolic syndrome (MS) is a clinical syndrome with multiple metabolic disorders. As the diagnostic criteria for MS still lacking of imaging laboratory method, this study aimed to explore the differences between healthy people and MS patients through infrared thermography (IRT). However, the observation region of the IRT image is uncertain, and the research tried to solve this problem with the help of knowledge mining technology. 43 MS participants were randomly included through a cross-sectional method, and 43 healthy participants were recruited through number matching. The IRT image of each participant was segmented into the region of interest (ROI) through the preprocessing method proposed in this research, and then the ROI features were granulated by the K-means algorithm to generate the formal background, and finally, the two formal background were separately built into a knowledge graph through the knowledge mining method based on the attribute partial order structure. The baseline data shows that there is no difference in age, gender, and height between the two groups (*P* > 0.05). The image preprocessing method can segment the IRT image into 18 ROI. Through the K-means method, each group of data can be separately established with a 43 × 36 formal background and generated a knowledge graph. It can be found through knowledge mining and independent-samples T test that the average temperature and maximum temperature difference between the chest and face of the two groups are statistically different (*P* < 0.01). IRT could reflect the difference between healthy people and MS people. The measurement regions were found by the method of knowledge mining on the premise of unknown. The method proposed in this paper may add a new imaging method for MS laboratory examinations, and at the same time, through knowledge mining, it can also expand a new idea for clinical research of IRT.

## Introduction

MS is a clinical syndrome with multiple metabolic disorders, mainly manifested as hyperglycemia, dyslipidemia, hypertension and obesity^1^. The prevalence is high, and the epidemic trend is becoming more serious. The prevalence was as high as 34.7% among people over the age of 18 in the United States from 2011 to 2016^2^. Evidence shows that MS significantly promotes the occurrence and development of cardiovascular and cerebrovascular diseases, and is an important risk factor for type 2 diabetes and COVID-19^3^. Compared with non-MS patients, the risk of cardiovascular and cerebrovascular diseases in MS patients increases 2 times, and the risk of all-cause mortality increases 1.5 times^4^.

Internationally, the clinical diagnosis and evaluation methods of MS are inconsistent, but it usually means that MS can be diagnosed with more than 3 items such as hyperglycemia, hyperlipidemia, obesity, and positive oral glucose tolerance test^5^. After decades of development, MS diagnostic methods still lack the participation of imaging laboratory examination technology. Therefore, if a fast, accurate, and radiation-free imaging inspection method can be established, it will add new perspectives and methods to researchers' understanding of MS.

With the advancement of IRT, it has made a lot of research results in clinical research, mainly involving tumor screening, pain location, motion monitoring and other fields, which belong to functional inspection technology^6^. The clinical application principle of IRT is to collect the skin temperature of the whole body or local area of the human body to infer the energy metabolism state of the subcutaneous blood circulation, tissues or organs^7^. Inflammatory changes in the subcutaneous tissue will increase the local skin temperature, and fat thickening or blood circulation will reduce the local skin temperature^8^. Yavuz et al.^9^ explained the reason for the elevated temperature of the diabetic foot through experiments, and pointed out that IRT will add a new tool for the evaluation of the severity of diabetic foot. Shilco et al.^10^ pointed out that IRT can accurately detect changes on the skin temperature of the relevant area in the pre-onset period of many types of diseases, which provides an important evaluation method for disease screening.

From the above analysis, it can be known that IRT technology can reflect the information of human skin temperature distribution, and MS is a systemic metabolic disease, which can theoretically cause changes in human skin temperature characteristics, but it is unknown which areas will change. Therefore, accurately finding out the target area of the measurement is a key issue that we urgently need to solve. For this reason, we try to use the knowledge mining technology in engineering research to help us find the area where the skin temperature of MS patients has changed.

Knowledge mining is an important research direction based on mathematics and popular in the fields of artificial intelligence and machine learning. APOS is a knowledge mining visualization technology proposed by our team. At present, the APOS has obtained many research results in the fields of Traditional Chinese Medicine, semantic mining, artificial intelligence and so on^11–13^. This research will use the IRT data of MS and healthy people as the carrier, try to mine the data through the theory, find the rules between the data, and verify the mining rules through statistical methods.

## Experiment and methods

### Study design

**Figure 1:**
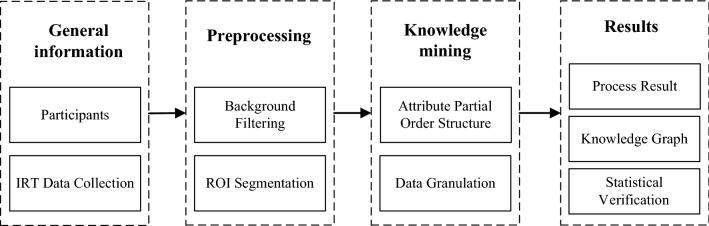
The design of this study.

The design of this study is shown in Fig. 1, including general information, pretreatments, knowledge mining, and results. After the participant IRT data is preprocessed, the image can be segmented into ROI sets. After the temperature characteristics in the ROI are granulated, the knowledge graph can be generated through APOS. After the knowledge mining of the control group and the MS group, the skin temperature distribution of the two groups can be obtained, and finally the mining results are verified by statistical methods. This research passed the review of the Ethics Committee of Dongzhimen Hospital in Beijing University of Chinese Medicine (No. DZMEC-KY-2020-102), and conducted in accordance with the Declaration of Helsinki.

### General information

#### Participants

Participants in this study came from the Physical Examination Center of the International Department of Dongzhimen Hospital, Beijing University of Chinese Medicine, and all participants signed the informed consent form. Participants did not receive any financial incentive during the trial, but all examinations were free of charge.

#### Inclusion criteria

1. The diagnostic of the participant should comply with MS diagnostic standards (Detailed diagnostic criteria are written in the supplementary materials section 1);
2. The age of the participant was greater than 18 years old;
3. The participant agreed to participate in this study.

#### Exclusion criteria

1. Women during pregnancy or lactation;
2. Those with a history of cardiovascular and cerebrovascular diseases, severe liver and kidney insufficiency, blood diseases, severe trauma or major surgery;
3. Diseases with metabolic abnormalities as the main manifestation, such as hyperthyroidism, hypothyroidism, acute thyroiditis, hypovolemia, etc.;
4. Participants have obvious allergic rash on the skin;
5. Those who have a history of mental illness or who do not agree to participate in this research.

#### IRT data collection

Due to the high sensitivity of IRT to temperature, it is prone to interference during data acquisition. This study refers to the consensus statement on human skin temperature measurement proposed in the literature^14^.

1. The individual data of all participants were recorded in the data table;
2. Participants had no alcohol, smoking, caffeine, large meals, ointments, cosmetics, and showers in the four hours before the images were collected;
3. Participants did not exercise vigorously within two hours before the image was collected;
4. The ambient temperature was 25 ± 1℃, and the relative humidity of the air was 40–50%;
5. There was no interfering heat source in the IRT field of view, and no obvious air flow;
6. The equipment is HIR-2000A cabin type IRT produced by Beijing Yuetian Optoelectronics Technology Co., Ltd. The thermal sensitivity was 0.05℃ at 30℃, the spectral range was 7.5–13 μm, the image pixel size was 256 × 336, the spatial resolution was 0.9mrad, and the acquisition frequency was 9 Hz.
7. The IRT collection environment is a thermostatic chamber, and all participants are in the same space to ensure that the environmental baseline is consistent;
8. Power on the equipment half an hour before data collection to keep the equipment in a steady state;
9. The vertical distance between the participant and the lens is 2.5 m;
10. The center line of the lens field of view is perpendicular to the human body through the liftable pan/tilt;
11. The emissivity of the detector was set to 0.98 (default);
12. The time for all participants to collect images is 9:00–11:00 am;
13. All participants maintained the same standing posture, with their arms drooping naturally, palms forward, arms and legs slightly extended;
14. Before collecting images, participants remove their clothes and accessories, and rest for 15 min in the thermostatic chamber;
15. The IRT data processing software is independently developed by our team. The data save format is a “*.dat” file, which contains all the temperature values of any space coordinates in the field of view, and is filled in a 16-bit integer data format.

### Preprocessing

The preprocessing include the background filtering method and the ROI segmentation method based on anchor point positioning. The purpose of background filtering is to filter out all the temperature information except the human body information in the image, so as to facilitate the automatic calculation of boundary coordinates during the ROI segmentation process. The purpose of ROI segmentation is to obtain the sub-domain information of the human skin temperature distribution, which is convenient to find the different regions of different groups of people through the method of knowledge mining.

#### Background filtering

IRT is a pseudo-color image, and the color channel of the image can be changed by adjusting the temperature forming width (tfw), temperature forming starting point (tfs), and palette^15^. Therefore, the conventional background filtering method based on color channels is not suitable for processing IRT.

**Figure 2:**
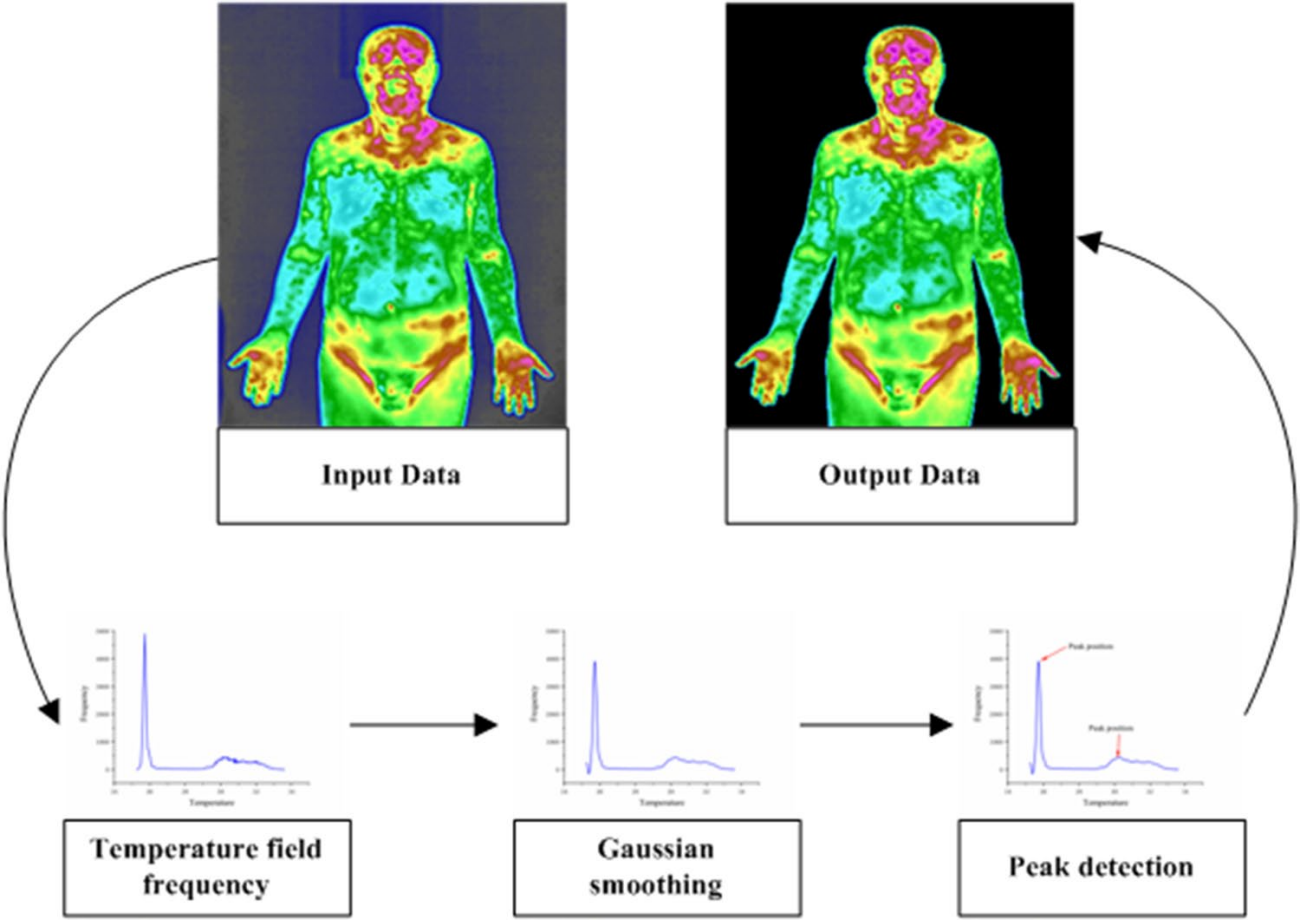
Flow chart of bimodal background filtering method.

This paper proposes a bimodal background filtering method based on the temperature field distribution, which can filter IRT background succinctly and quickly, and retain the effective data of human skin IRT. The flow chart of the algorithm is shown in Fig. 2, and the specific operation flow is shown as follows:

1. Extracting the IRT temperature field data to be processed as input;
2. Calculating the distribution frequency of each temperature value in the field and drawing a curve;
3. Performing gaussian smooth filtering on the curve, and seting the operator size to 5;
4. Detecting and locating the first two peaks of the processed curve;
5. Calculating the median between the two peaks as the threshold to segment the image background.

#### ROI segmentation

The purpose of this study is to explore the rule of skin temperature distribution on different parts, but there are individual differences in the participants on height and weight, and it is difficult to perform rapid segmentation through image processing. A semi-automatic region segmentation method based on anchor marking was proposed in this study. This process required the operator to manually mark the anchor points, and then generate ROI through mathematical calculation and image processing to complete the image segmentation.

**Figure 3:**
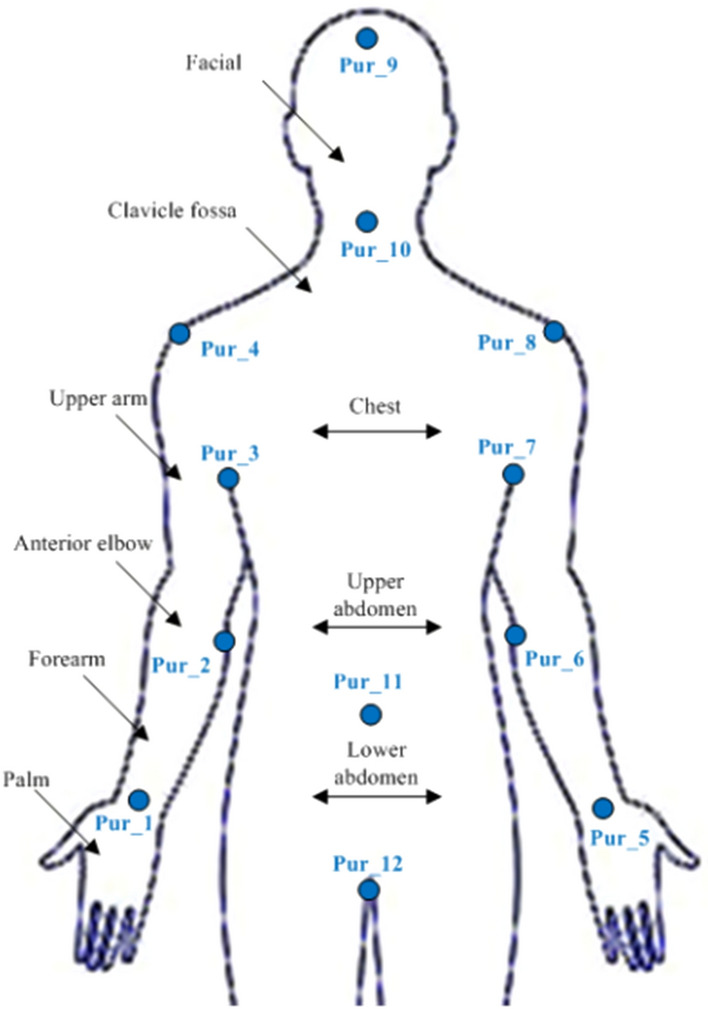
The region segmentation target.

Determining the measurement targets are the premise of segmenting the ROI of the people IRT. In this study, the human image was divided into 18 ROI of symmetry according to the midline, and corresponding to 12 anchor points, as shown in Fig. 3. Each anchor point corresponds to the body surface marking part of the participant as shown in Table 1. According to the information of 12 anchor points, the vertices of each ROI can be quickly calculated. The comparison relationship between ROI labels and anchor points is shown in Table 2.

**Table 1:** A comparison table of Purhor information and body surface marks.

Pur_1	Pur _2	Pur_3	Pur_4	Pur_5	Pur_6	Pur_7	Pur_8	Pur_9	Pur_10	Pur_11	Pur_12
Right wrist	Medial right elbow	Right armpit	Right shoulder	Left wrist	Medial left elbow	Left armpit	Left shoulder	Forehead	Chin	Belly-button	Crotch

**Table 2:** The relationship between theROI labels and the Purhor points.

Labels	Body parts	Purhors
ROI1	Right palm	Pur_1
ROI2	Right forearm	Pur_1, Pur_2
ROI3	Right anterior elbow	Pur_2
ROI4	Right upper arm	Pur_2, Pur_3, Pur_4
ROI5	Left palm	Pur_5
ROI6	Left forearm	Pur_5, Pur_6
ROI7	Leftt anterior elbow	Pur_6
ROI8	Left upper arm	Pur_6, Pur_7, Pur_8
ROI9	Right face	Pur_9, Pur_10
ROI10	Left face	Pur_9, Pur_10
ROI11	Right clavicle fossa	Pur_4, Pur_8, Pur_10
ROI12	Left clavicle fossa	Pur_4, Pur_8, Pur_10
ROI13	Right chest	Pur_3, Pur_4, Pur_7, Pur_8, Pur_11
ROI14	Left chest	Pur_3, Pur_4, Pur_7, Pur_8, Pur_11
ROI15	Right upper abdomen	Pur_4, Pur_8, Pur_11
ROI16	Left upper abdomen	Pur_4, Pur_8, Pur_11
ROI17	Right lower abdomen	Pur_11, Pur_12
ROI18	Left lower abdomen	Pur_11, Pur_12

After background segmentation, the background had been set to 0 °C, so the boundaries of each ROI could be found through row and column traversal according to the anchor points. The circumscribed quadrilateral area of the target region could be extracted, and the background information contained in the quadrilateral could be excluded through later calculation. The key to the extraction of the quadrilateral region is the acquisition of the coordinates of its four vertices. Different regions have different calculation methods. The ROI vertex coordinates are mainly calculated based on the same-size scale.The specific calculation method can be found in the supplementary materials section 2.

### Knowledge mining method based on APOS

#### The basic principle of APOS

APOS is proposed on the basis of order theory, granular computing, and formal concept analysis. Its main purpose is to visually analyze and mine the knowledge structure between attributes and objects in data tables^16^. The generation principle of APOS as follows:

1. The attribute nodes with more coverage objects are higher in the hierarchy, and the attribute nodes with fewer coverage objects are lower in the hierarchy;
2. The same or similar objects are close to each other, and different objects are far away from each other;
3. The higher-level attribute nodes are more universal and the lower-level attribute nodes are more specific;
4. Each branch is a collection of attributes of an object.

This article does not give a detailed introduction to the specific generation method. The supplementary material provides a simple explanation of the method through a simple example (section 3). For the specific generation code, please refer to the literature^17,18^.

#### IRT data granulation method

The formal background is a binarized N × S data table composed of attributes and objects. Each object corresponds to N attributes, and each attribute corresponds to S objects. The formal background also is a binary data table, and the generation of APOS is based on the table. Therefore, how to transform the image features of the IRT into the formal background, will be the key point to this research.

**Figure 4:**
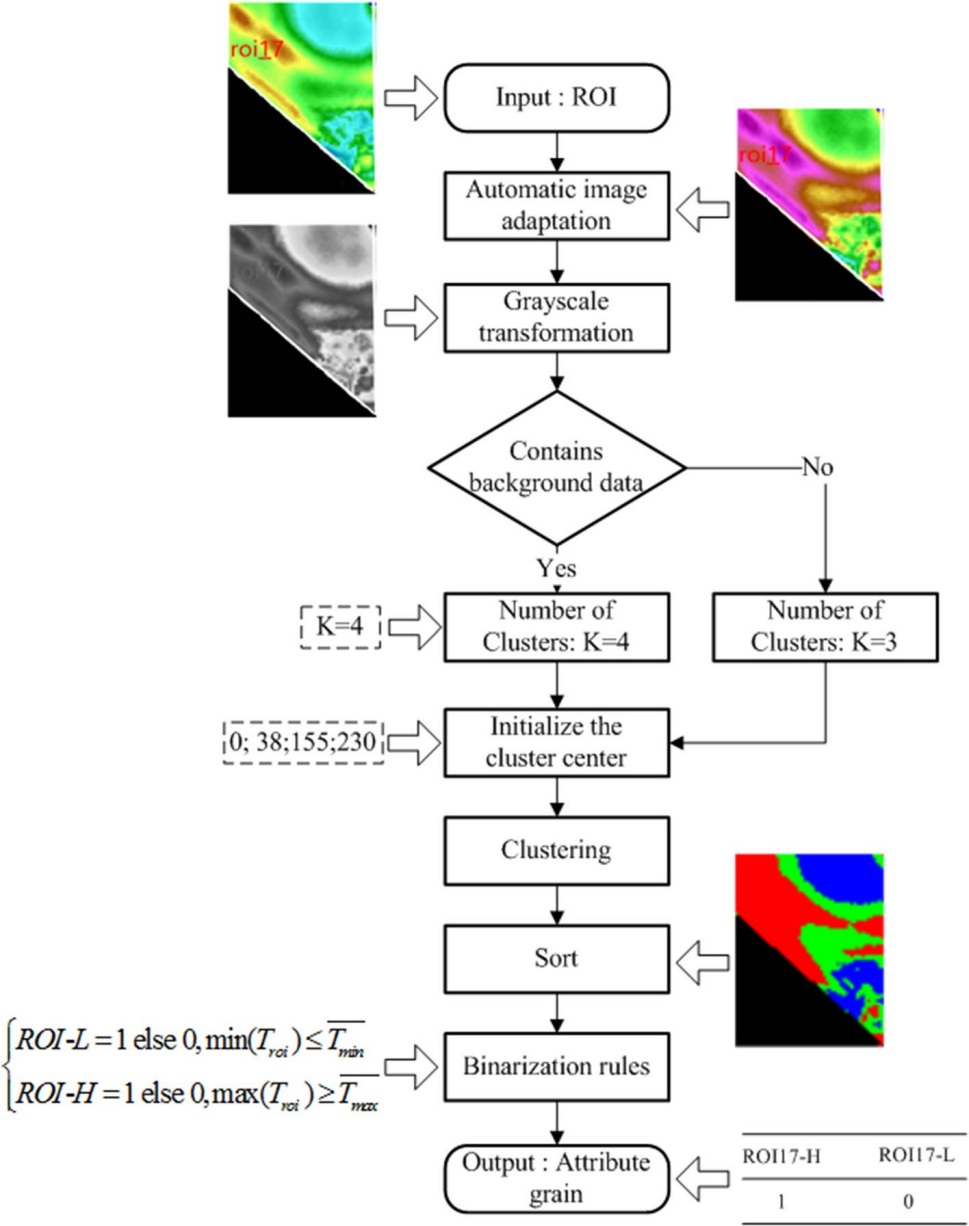
Flow chart of data granulation.

This paper proposed a regional feature granulation method based on K-Means.The flow chart is shown in Fig. 4. The core idea of this method was to cluster the ROI data into three types: high temperature, medium temperature, and low temperature, and then distinguish the high and low temperature according to the clustering results and the temperature features of all ROI. A formal background with two attributes of high temperature and low temperature should be generated in each ROI.

K-Means is a commonly used unsupervised clustering algorithm, which has obvious advantages, but also has disadvantages, such as: sensitivity to noise and discrete points; the hyperparameter K must be determined in advance; if the center point is initialized randomly, each clustering result is inconsistent^19^. Therefore, the design goal of this algorithm is to reduce the impact of the above disadvantages of the clustering results as much as possible.

Different tfw and tfs can show the same data as different images. The difference in the discrete points of the image is particularly obvious, which has a great impact on the clustering of K-Means. Therefore, the drawing rule should be standardized, and the parameters of all ROI of each participant must be consistent. To solve this problem, this paper proposed an adaptive image adjustment method, which was based on temperature data. The tfw usually be set to 10℃ in medical IRT research^15^, and in this study, tfw was initialized to 10℃, and tfs was set to the difference between the maximum temperature and tfw. At the same time, the grayscale of the standardized image could further reduce the impact of the palette on the data and reduce the computational complexity.

The goal of the algorithm is to extract the high-temperature attribute and the low-temperature attribute in the ROI. As some of the extracted ROI contain background data, but some do not. The background has an influence on the number of classifications, when there was background data in the ROI, the super parameter K was set to 4, otherwise the K was set to 3, which could solve the problem of preset K in the K-Means algorithm.

The random center point method is often used in the initialization of K-Means, but this method may result in inconsistent clustering results each time, and the robustness is poor. In order to solve this problem, this study can initialize the center point according to the distribution features of IRT temperature data to ensure the consistency of the clustering results of all ROI in the same image. When K was 4, the four center points were set to 0, non-zero minimum value, non-zero average value, and non-zero maximum value, and when K was 3, the three center points were set to the minimum, average and maximum value. This method directly enhances the robustness of the algorithm.

After K-Means clustering, each ROI can be divided into four clusters: high temperature, medium temperature, low temperature and background. When the temperature of the high-temperature cluster in the ROI was higher than the average of the high-temperature clusters in all regions, it means that the high-temperature attribute in the region was dominant. Similarly, whether the low-temperature attribute was dominant could also be judged. This is the process of converting the image data into qualitative data. The specific discriminant arithmetic model is shown in formula (1):

1 $$ \left\{ \begin{gathered} ROI{ - }L = 1\;{\text{else }}\;{0},\;\min (T_{roi} ) \le \overline{{T_{min} }} \hfill \\ ROI{ - }H = 1\;{\text{else}}\;{ 0},\;\max (T_{roi} ) \ge \overline{{T_{max} }} \hfill \\ \end{gathered} \right. $$

where *ROI-L* represents the low-temperature attribute of the ROI, *ROI-H* represents the high-temperature attributeof the ROI region, *T*_*roi*_ represents the average temperature set of the non-zero clusters obtained by K-Means of the target region, $\overline{{T_{min} }}$ represents the average temperature of the smallest non-zero cluster in all ROI of the whole body, and $\overline{{T_{max} }}$ represents the average temperature of the largest cluster in all ROIs of the whole body.

The human IRT segmentation method described in section 2.3 was used to divide the image into 18 ROI. Combined with the data granulation method, 18 ROI could be granulated into 2 × 18 attribute sets, where each ROI corresponded to two attributes of *ROI-L* and *ROI-H*. The two formal backgrounds of this study would be constructed corresponding to the control group and the MS group, with the same number of data objects and the same scale of knowledge graph, and the qualitative description of the IRT image can be realized, and a knowledge graph can be constructed to facilitate subsequent knowledge mining.

### Statistics

Continuous variables are expressed as the mean (standard deviation), and categorical variables are expressed as numbers and percentages.The continuous variables of baseline characteristics were evaluated with ANOVA, and the categorical variables were evaluated with χ^2^ test. For the characteristics of the target area obtained through knowledge mining were evaluated with independent-samples T test. Analyses were performed with SPSS (IBM SPSS Statistics, New York) Version 26 with *P* < 0.05 considered significant.

## Results and analysis

### Participants and baseline

The research time node is May 7, 2020 to May 7, 2021. 43 MS participants were recruited (affected by COVID-19), marked as MS Group. In order to reduce the difference in the data size of the knowledge graph between groups, the sample size of healthy participants was determined to be 43 and randomly recruited, marked as Control Group. The participant demographic and baseline characteristics were shown in Table 3.

**Table 3:** Participant demographic and baseline characteristics.

Characteristic	MS group (N = 43)	Control group (N = 43)	*P*
Age, mean ± SD, years	38.95 (7.074)	37.70 (9.498)	0.489
Female, no(%)	21 (48.84)	20 (46.51)	0.832
Height, mean (SD), cm	169.85 (8.67)	168.46 (6.89)	0.411
Female	165.99 (8.15)	163.51 (4.87)	0.248
Male	173.55 (7.60)	172.76 (5.34)	0.690
Weight, mean (SD), kg	81.70 (14.98)	62.33 (7.88)	≤ 0.001**
Female	75.24 (16.34)	56.62 (6.04)	≤ 0.001**
Male	87.87 (10.63)	67.31 (5.60)	≤ 0.001**
BMI, mean (SD), kg/m^2^	28.19 (4.12)	21.94 (2.22)	≤ 0.001**
Waistline,mean (SD), cm	96.87 (9.31)	75.60 (7.10)	≤ 0.001**
SBP, mean (SD), mm·Hg	131.84 (18.24)	112.98 (8.81)	≤ 0.001**
DBP,mean (SD), mm·Hg	83.19 (14.55)	70.26 (6.89)	≤ 0.001**
TG,mean (SD), mmol/L	2.69 (1.74)	0.96 (0.27)	≤ 0.001**
FBG, mean (SD), mmol/L	7.36 (2.84)	5.14 (0.28)	≤ 0.001**
HDL-C,mean (SD), mmol/L	1.10 (0.27)	1.45 (0.24)	≤ 0.001**

*BMI* Body Mass Index (calculated as weight in kilograms divided by height in meters squared), *SBP* systolic blood pressure, *DBP* diastolic blood pressure, *TG* triglyceride, *FBG* fasting blood glucose, *HDL-C* high density liptein cholesterol.

**Significant difference.

### The ROI segmentation results of IRT data

**Figure 5:**
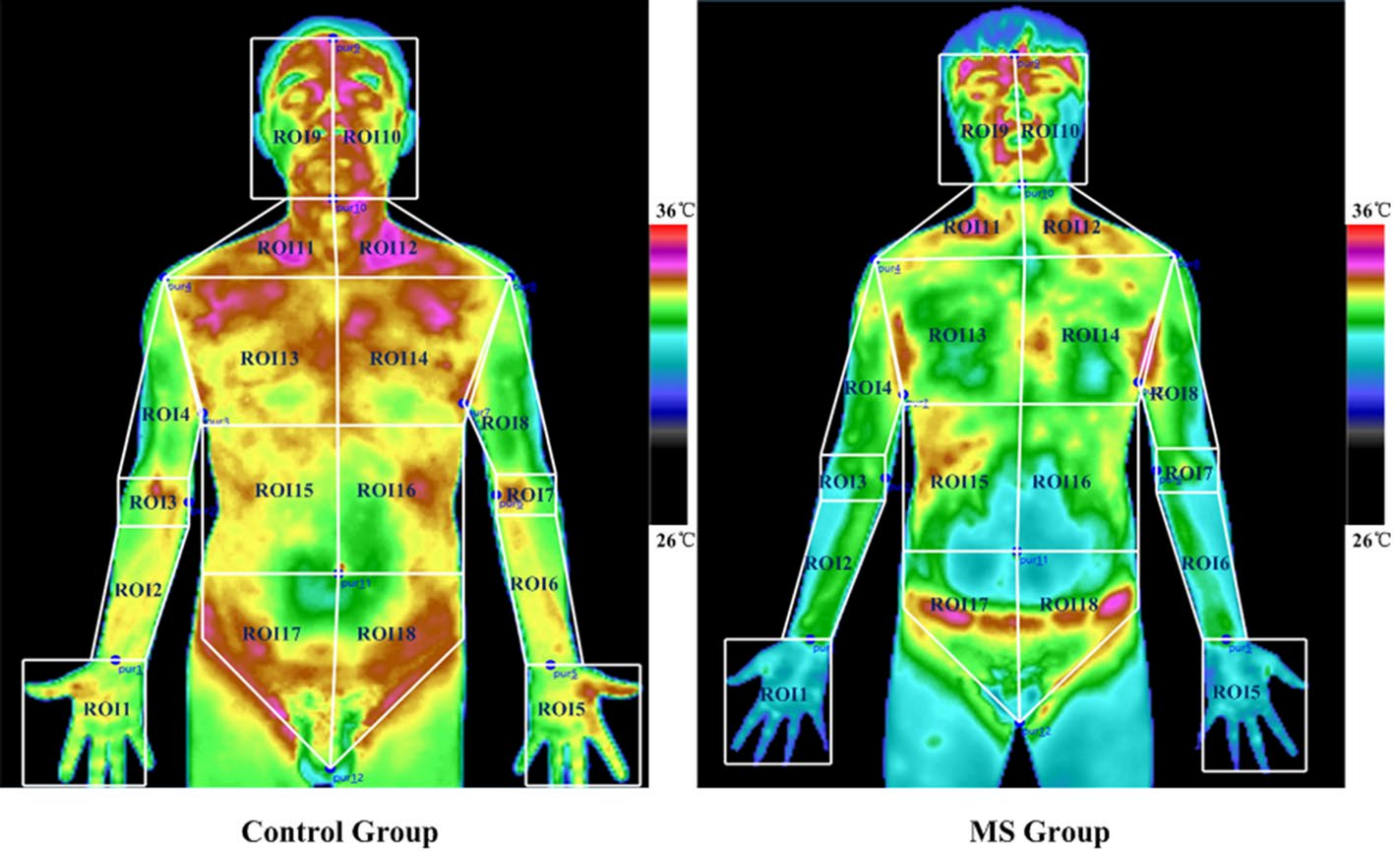
ROI segmentation results of the IRT data.

Through the segmentation method proposed in this research, the participant’s IRT data can be segmented into 18 ROI. The segmentation result is shown in Fig. 5. Each ROI is composed of quadrilaterals, and some ROI contain background data , but after the background filtering operation, these data are all set to 0, which does not affect the calculation of subsequent temperature characteristics.

### IRT feature granulation and knowledge graph generation results

After processing by the K-Means clustering algorithm, each ROI has generated two attributes of high temperature and low temperature, a total of 36. The number of participants in each group is 43. Due to the large amount of background data in the binary form generated through feature granulation, we have written this part of the result into the supplementary materials for readers to view (section 4).

**Figure 6:**
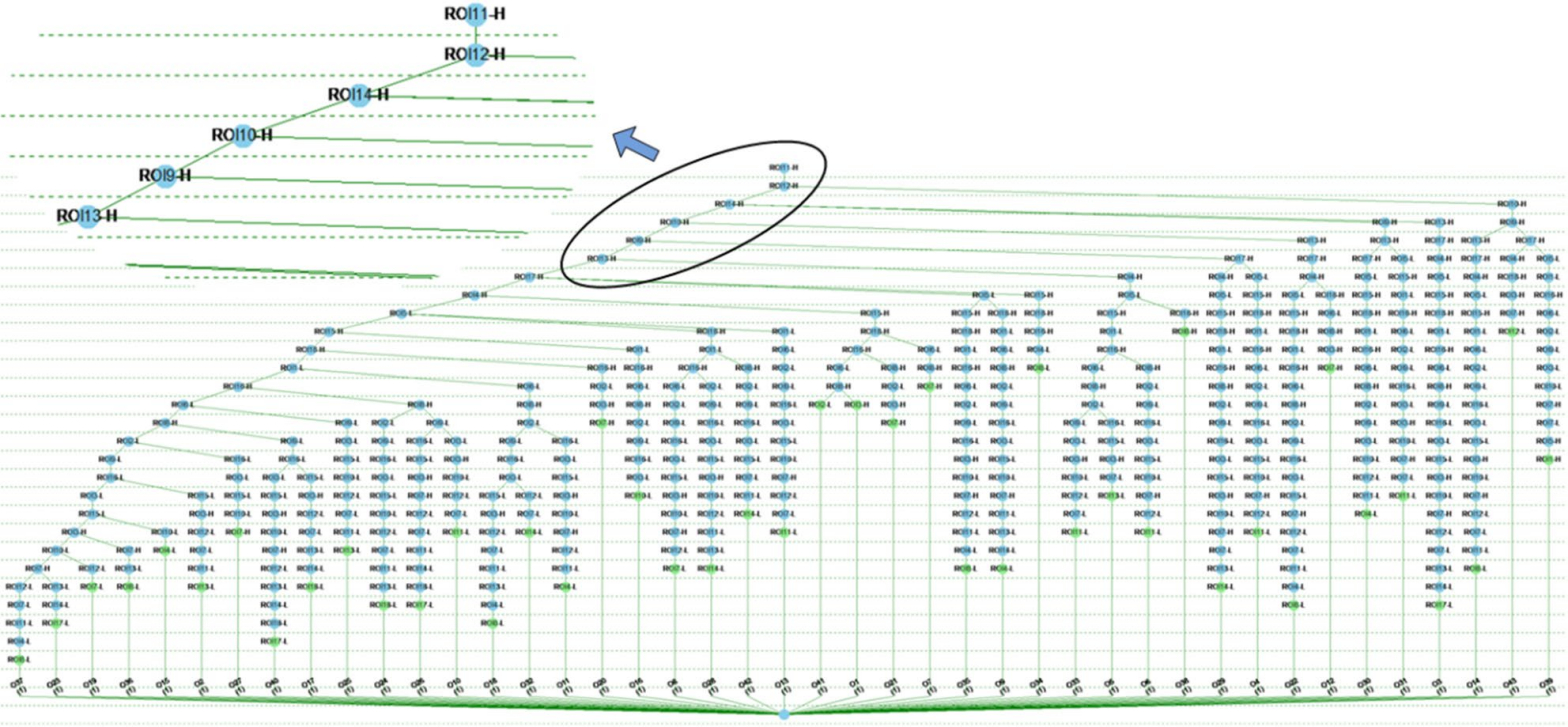
APOS diagram of IRT characteristics of control group.

**Figure 7:**
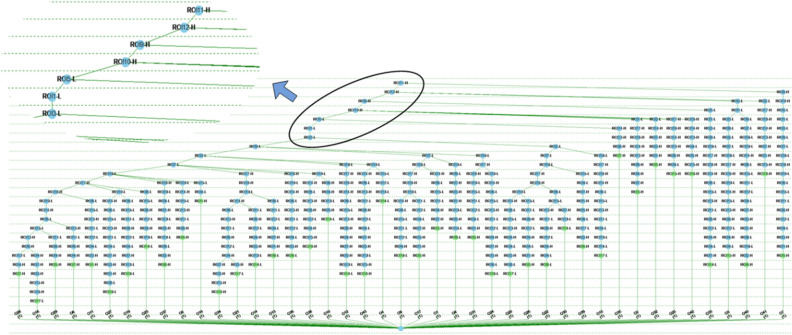
APOS diagram of IRT characteristics of MS group.

Through the method proposed above, two groups of the participants respectively generated two APOS diagrams as shown in Figs. 6 and 7 (High-Definition images have been uploaded to the supplementary materials section 5). By observing the distribution of attribute nodes in each layer of the knowledge graph from top to bottom, we can obtain the following rules (knowledge):

1. The high-temperature attribute nodes in the four regions ROI9, ROI10, ROI11, and ROI12 (clavicle fossa, face) are at a higher level, and it can be inferred that the IRT images of the control group and the MS group have the same rule.
2. The high-temperature attribute nodes of ROI13 and ROI14 (chest) of healthy people are at a higher level, and these two attribute nodes of MS group are lower, which can infer that the temperature features of the chest between the two groups may have difference.
3. The IRT features of the participants can be distinguished by different node sequences on the APOS diagram, which is expressed as the attribute set of each branch corresponds to only one participant. If the sample is large enough, a sufficiently complete rule base can be generated in theory, which may realize automatic classification of new samples.

Since the basal body temperature of each participant is affected by factors such as age, sex, day and night, the skin temperature of the participant is directly used for statistical analysis, which will greatly affect the experimental results^20^. To solve this problem, the clinical application of IRT often uses the temperature difference between different regions of the participant as an important evaluation parameter, which can reduce the impact of the above factors. So, according to the obtained rules, it can be further inferred that the temperature difference between the face and chest of the two groups may be significantly different.

### Statistical results

**Figure 8:**
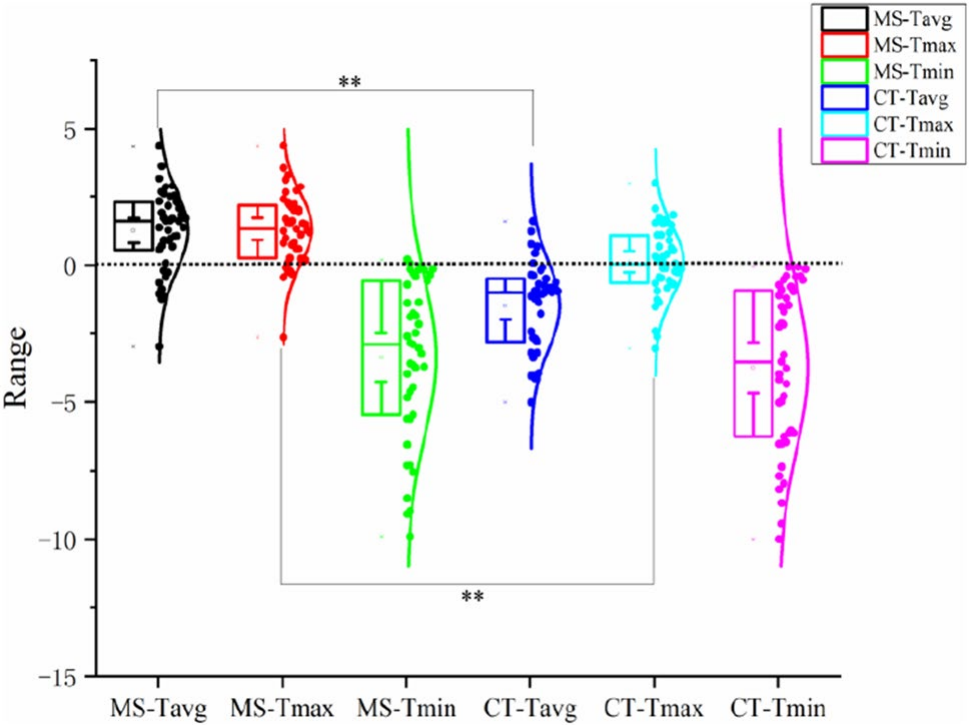
Distribution map of temperature difference between face and chest.

To verify the above inference, the temperature features of the face and chest of the participants were extracted, and the temperature difference was calculated, as shown in Fig. 8. The data distribution of the temperature difference of the average (△Tavg), maximum (△Tmax) and minimum (△Tmin) were displayed in the form of a box plot. From the data distribution, it can be found that △Tavg and △Tmax may be different between the two groups of people, but the difference between △Tmin is not obvious.

The statistical results are shown in Table 4. △Tavg and △Tmax have significant statistical differences, but △Tmin has no statistical difference, which is consistent with the proposed inference results.

**Table 4:** The result of independent-samples T test.

Temperature difference	t	df	Sig. (2-tailed)	Mean difference	Std.Error Difference	95% Confidence interval of the difference	
						Lower	Upper
△Tavg	8.235	83.4	≤ 0.001**	2.74	0.33	2.08	3.40
△Tmax	4.299	84	≤ 0.001**	1.19	0.28	0.64	1.75
△Tmin	0.589	83.889	0.588	0.37	0.63	-0.89	1.63

**Significant difference.

In this study, the sample size could not be estimated, as there were no relevant literature, no pre-experiments, and the observation target area and feature values were unknown when recruiting participants. According to the above statistical results, we can calculate the power of this study based on the mean of △Tavg or △Tmax, the standard deviation and the bilateral α level of 0.05, and the results are all greater than 0.9. We can think that the recruited 43 samples in each group can support this study.

## Discussion

It can be seen from the research results of this paper that the IRT of MS people and healthy people are different, but it is difficult to obtain meaningful measurement targets directly through human observation. Therefore, to obtain more valuable information from images, knowledge mining technology is needed.

The temperature distribution of human skin can vary from 4 to 6 °C. Normally, due to the interference of physiological structure, high temperature features appear in depressed areas such as the armpits, clavicle fossa, and the inside of the elbow. Low temperature features will appear in the protruding areas such as the tip of the nose, nipple, and the outside of the elbow^21^. It can be seen from the APOS diagram of the two groups that most of the highest-level nodes are high-temperature properties in the clavicle fossa area, which also matches the human skin temperature distribution rule mentioned in the literature^21^.

The chest IRT features of the two groups have different levels on the APOS diagram. After statistical verification, the temperature difference have significant differences in between the chest and face.This result is obtained without the intervention of clinical prior knowledge and is an objective result. MS patients have characteristics of high blood sugar, hypertension, high triglycerides. Study has shown that the high blood sugar can cause platelet dysfunction, which in turn leads to hypercoagulable products. Under the action of a variety of factors that promote platelet adhesion to the vascular endothelium, and the vascular endothelial cell damage increases^22^. At the same time, the scope of vascular damage caused by high blood sugar is gradually expanded, which gradually increases the tendency of platelet aggregation reaction, and these are also a prerequisite for subsequent vascular lesions. In addition, dyslipidemia will cause the intimal damage of arteries and blood vessels to expand, and the continuous accumulation of platelets and other substances will form atherosclerotic plaques that protrude into the lumen of the arteries, cause hemodynamic changes, and stenosis of arterial blood vessels, and eventually affect blood circulation^23^. Therefore, under the action of a series of related factors such as hypertension, hyperglycemia and hyperlipidemia, the hemodynamic changes inside the blood vessels are obvious, the blood in the hypercoagulable state causes the venous blood to return back to slow down. Atherosclerotic stenosis can increase the internal blood flow velocity, which will cause the blood flow energy inside the arterial blood vessels to increase accordingly. The hardening and distortion of arteries in patients with hypertension destroy the complete dual temperature regulation and balance mechanism in the center and the periphery, which can also lead to abnormal temperature regulation. The vascular network of the human face is densely distributed, and the increase in blood flow energy and the slowing of venous blood return caused by abnormal blood circulation in MS patients may cause the facial heat features to be more obvious. Through the above analysis, we can roughly infer: the large temperature difference between face and chest of the two groups is more likely due to the abnormal temperature distribution of the face, rather than the chest.

In the clinical research of IRT, most of the measurement regions are determined by prior knowledge, but this will be limited by prior knowledge. One of the advantages of IRT is that it can observe the skin temperature distribution information of the whole body. If the measurement regions are defined in advance, it seems to reduce the actual value of IRT. Therefore, discovering the rule and knowledge in the data is an important way to take advantage of IRT technology. This study has made a preliminary attempt on it in the form of knowledge mining. In the following research, we can continue to mine more knowledge from the knowledge graph, and also may increase the data dimension of the knowledge graph and integrate diverse parameters to obtain more knowledge concepts with clinical application value.

Limitations and advantages:

For the participants, there may be other confounding diseases that interfere with the IRT and thus affect the results. In this case, during the research process, we strictly controlled the purity of the data through inclusion and exclusion criteria to ensure the accuracy of the results. However, this idea may be different from engineering, we did not consider the influence of more confounding factors on the final research results, and it is also true that clinical practice is prone to confounding of other unknown diseases among participants, so our method may be less robust.

The clinical data of our study were obtained with strict control of inclusion and exclusion criteria. For the participants with diseases that affect IRT data, we excluded them during the recruitment process, so the impact on the results can be avoided as much as possible. We also conducted a statistical test on the results, which greatly improved the accuracy of the research, and also established a scientific research basis for the follow-up research we are going to carry out.

In addition, our study applied the knowledge graph, but it is currently only used to mine regularities, find ROIs with research value for clinical researchers, and discuss them in combination with physiological effects. Participant disease prediction studies were not conducted. In fact, this knowledge graph method is also an important tool for our future research. We will increase the sample size, increase confounding factors, and improve its ability to identify MS.

In this study we have not yet identified the physiology of MS and IRT, which is what we are trying to solve. As a methodological study of MS with IRT, it may inspire new research ideas for readers. However, environmental information has a great impact on infrared thermography, and future research also needs to focus on this issue.

## Conclusion

The difference between MS and healthy people in IRT was discussed in this study. Under the premise that the observation region was not clear, we performed knowledge mining on the data through the theory of the APOS. According to the node information of the knowledge graph, it can be inferred that the temperature difference between the face and chest of the two groups may be significantly different. After discussion and statistical analysis of clinical knowledge, the rules were proved to be accurate, and these phenomena are explained through medical principles. This research may provide a new method for MS imaging evaluation, and through knowledge mining method, it also provide new ideas for the clinical research of the IRT.

## Supplementary Information

Supplementary Information.

## Data Availability

The IRT data set involved in this research is stored in a software system funded by our team. The image contains the private information of all participants. If it is shared directly, it will infringe the rights of the participants. For inquiries about data sharing, please send request at mibaohong12587@163.com.
