# Supplementary material for: An exploration of new methods for metabolic syndrome examination by infrared thermography and knowledge mining
Source: Sci Rep. 2022 Apr 16;12:6377. doi: 10.1038/s41598-022-10422-6 (PMC9012989; doi:10.1038/s41598-022-10422-6)
Supplement: Supplementary file 1 — Supplementary Information. [file 41598_2022_10422_MOESM1_ESM.docx]

**Supplementary materials**

**Section 1.MS diagnostic standards**

The MS diagnostic criteria was according to the IDF consensus worldwide definition of the metabolic syndrome, and the primary condition for the diagnosis of MS was that the waist circumference of men was greater than 90cm and that of women was greater than 80cm. Combine any 2 of the following 4 indicators to meet the MS diagnostic criteria:

(1) The level of triglyceride (TG) was increased (>1.7mmol/L), or had received corresponding treatment;

(2) The level of high-density lipoprotein cholesterol (HDL-C) decreased (male <1.03 mmol/L, female <1.3 mmol/L), or had received corresponding treatment;

(3) Increased blood pressure (systolic blood pressure ≥130 mmHg or diastolic blood pressure ≥85 mmHg), or had received corresponding treatment, or had previously been diagnosed with hypertension;

(4) Elevated fasting blood glucose (FBG) (≥5.6 mmol/L), or had received corresponding treatment, or had previously been diagnosed with type 2 diabetes.

**Section 2.The specific calculation method of ROI segmentation**

Since the 18 target regions are bilaterally symmetric, this paper takes the right side as an example to introduce, the same is true on the left side, and the calculation methods are as follows:

ROI1 corresponds to the position of the palm, and a quadrilateral position can be determined according to an anchor point of 'Pur_1'. Taking the ordinate of the anchor point as the upper edge of the quadrilateral, traversing down to the position of the fingertip as the lower edge of the quadrilateral, and taking the tip of the thumb and the inside of the wrist as the boundary of its side, and then taking the intersections of the four boundary lines as the vertexs of the quadrilateral.

ROI2 corresponds to the forearm, and the position of the quadrilateral is determined according to 'Pur_1' and 'Pur_2'. Calculate the elbow width marked as w through 'Pur_2', and the following offset w/3 is the upper boundary of ROI2, and the upper boundary of ROI1 is the lower boundary of ROI2. Traverse the two anchor points horizontally to obtain the inner and outer boundaries of the forearm, and connect the four vertices.

ROI3 corresponds to anterior elbow, which can be determined according to 'Pur_2'. Calculate the elbow width marked as w by 'Pur_2', and calculate the upper and lower boundaries of ROI3 respectively by the upper and lower offsets w/3, to locate the rectangular target region.

ROI4 corresponds to the upper arm, which can be determined according to 'Pur_2', 'Pur_3' and 'Pur_4'. Take the coordinates of the upper vertex of ROI3 as the two vertices of ROI4, and connect 'Pur_3' and 'Pur_4' to determine the quadrilateral region.

ROI9 corresponds to the face, which can be determined according to 'Pur_9' and 'Pur_10'. According to the two anchor points, the upper and lower boundaries and the inner boundary of the quadrilateral can be determined. The outer border can be found through horizontal and vertical traversal to find the ear part as the outer border, which can determine the quadrilateral region.

ROI11 corresponds to the clavicle fossa, which can be determined according to 'Pur_4', 'Pur_8', and 'Pur_10'. Traverse 'Pur_10' to determine the neck boundary as the upper boundary of ROI11, connect 'Pur_4' and 'Pur_8' as the lower boundary, connect 'Pur_10' and the midpoint of the lower boundary as the inner boundary, and connect 'Pur_4' and the neck boundary as the outer boundary to determine the quadrilateral region.

ROI13 corresponds to the chest, which can be determined according to 'Pur_3', 'Pur_4', 'Pur_7', 'Pur_8', 'Pur_11'. The lower boundary of ROI11 is taken as the upper boundary of ROI13. Calculate the vertical distance marked as h from the midpoint of 'Pur_4' and 'Pur_8' to 'Pur_11', take the position from the upper boundary downward h/2 as the lower boundary, use 'Pur_3' to calibrate the lower boundary width, and connect the vertices to determine the quadrilateral region.

ROI15 corresponds to the upper abdomen, which can be determined according to 'Pur_4', 'Pur_8' and 'Pur_11'. The lower boundary of ROI13 is taken as the upper boundary of ROI15. Determine the lower boundary according to 'Pur_11' with the width is the same as the upper boundary to determine the rectangular region.

ROI17 corresponds to the lower abdomen, which can be determined according to 'Pur_11', 'Pur_12'. The lower boundary of ROI15 is taken as the upper boundary of ROI17. Calculate the vertical distance of 'Pur_11' and 'Pur_12' marked as h1, and take the outer vertex of the upper boundary down h1/3 as the outer vertex of the lower boundary, and connect 'Pur_11' and 'Pur_12' to determine the polygon region.

**Section 3.The simple example of the APOS**

To clearly describe the APOS generation process, the qualitative data set Lives in water provided on the official website of the formal concept analysis theory FCA Homepage [1] is taken as an example, as shown in Table 1.The APOS diagram generated by the data set is shown in Figure 1.

Table 1 Form background of lives in water

|  | a | b | c | d | e | f | g | h | i |
| --- | --- | --- | --- | --- | --- | --- | --- | --- | --- |
| 1 | ☆ | ☆ |  |  |  |  | ☆ |  |  |
| 2 | ☆ | ☆ |  |  |  |  | ☆ | ☆ |  |
| 3 | ☆ | ☆ | ☆ |  |  |  | ☆ | ☆ |  |
| 4 | ☆ |  | ☆ |  |  |  | ☆ | ☆ | ☆ |
| 5 | ☆ | ☆ |  | ☆ |  | ☆ |  |  |  |
| 6 | ☆ | ☆ | ☆ | ☆ |  | ☆ |  |  |  |
| 7 | ☆ |  | ☆ | ☆ | ☆ |  |  |  |  |
| 8 | ☆ |  | ☆ | ☆ |  | ☆ |  |  |  |

Note:1:fish leech, 2:bream, 3:frog, 4:waterweeds, 5:reed, 6:bean, 7:corn; a: needs water to live, b: lives in water, c: lives on land, d: needs chlorophyll, e: dicotyledon, f: monocotyledon, g: can move, h:has limbs, i:breast feeds; ☆: yes


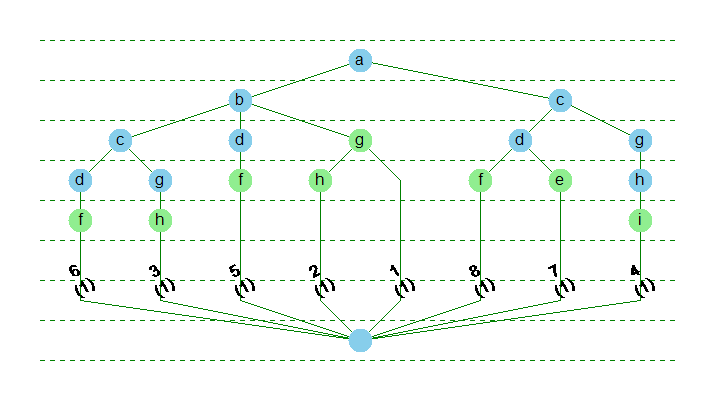


Figure 1 The APOS of lives in water

It can be seen from the figure that the attribute 'a' is located at the top layer, and it has the most generality. The label of 'a' is: needs water to live, and it can be interpreted as all objects in this background need water. According to this top-down traversal method, it can be found that the nodes on the second layer are 'b' and 'c', which correspond to lives in water and lives on land, and the objects in this formal background can be divided into water life and land life. The further down, the fewer objects covered by the attribute, the stronger the specificity of the node, until all the objects are distinguished. In the end, all the nodes of each branch correspond to a set of attributes possessed by an object.

**Section 4.Formal background of the two groups**


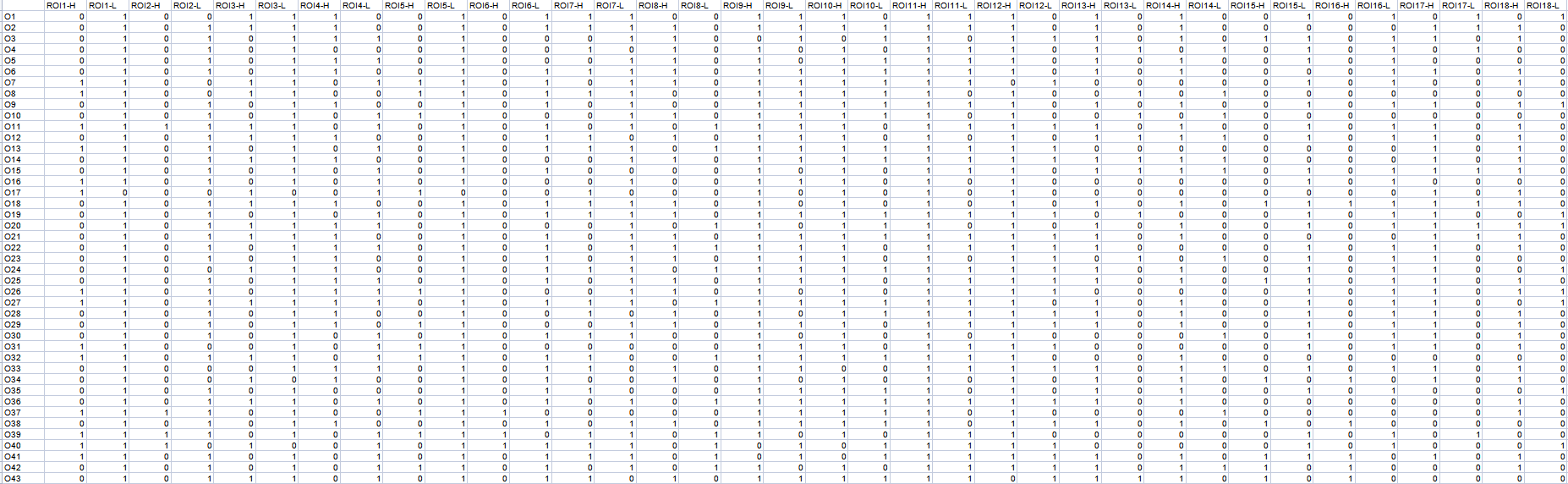


Figure 2 The formal background of the MS Group


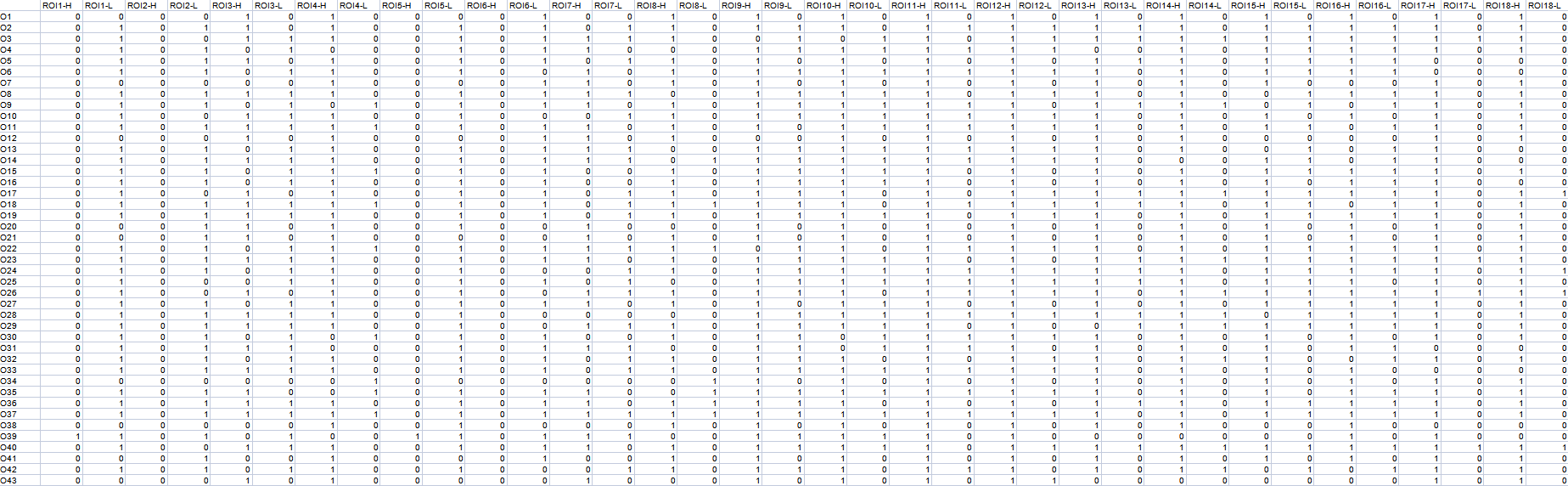


Figure 3 The formal background of the Control Group

**Section 5. High-definition(HD) images for the Figure 6 and Figure 7**

The HD images are as follows, and readers can zoom in and out by themselves.


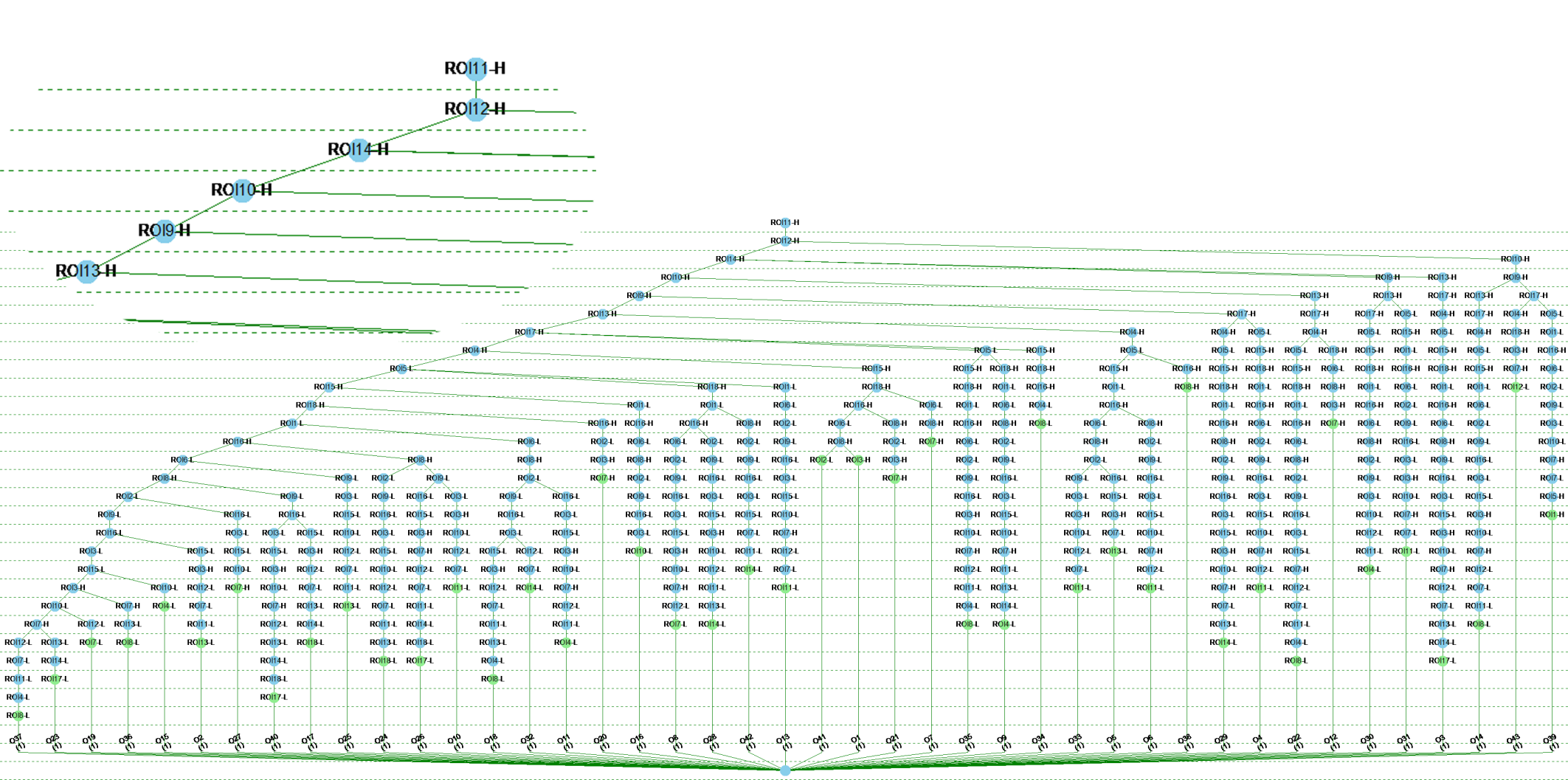


Figure 6


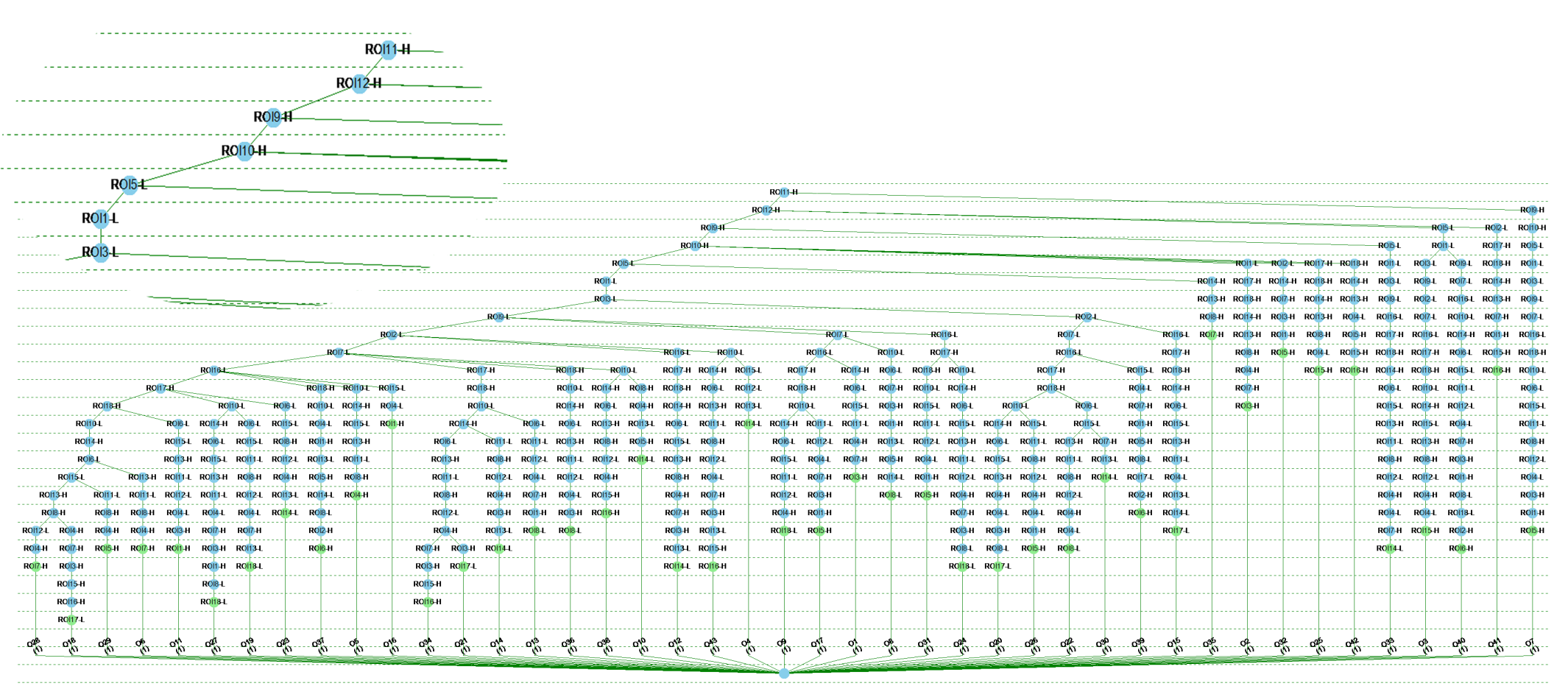


Figure 7

# References

[1] Formal Concept Homepage. http://www.upriss.org.uk/fca/examples.html[EB/OL]. 2016.
